# The detection of prostate cancer based on ultrasound RF signal

**DOI:** 10.3389/fonc.2022.946965

**Published:** 2022-12-12

**Authors:** Tianlei Xiao, Weiwei Shen, Qingming Wang, Guoqing Wu, Jinhua Yu, Ligang Cui

**Affiliations:** ^1^ School of Information Science and Technology, Fudan University, Shanghai, China; ^2^ Department of Ultrasound, Peking University Third Hospital, Beijing, China

**Keywords:** prostate cancer, RF time series, feature map, spectral feature, radiomics

## Abstract

**Objective:**

The diagnosis of prostate cancer has been a challenging task. Compared with traditional diagnosis methods, the radiofrequency (RF) signal is not only non-invasive but also rich in microscopic lesion information. This paper proposes a novel and accurate method for detecting prostate cancer based on the ultrasound RF signal.

**Method:**

Our approach is based on low-dimensional features in the frequency domain and high-throughput features in the spatial domain. The whole process could be divided into two parts: first, we calculate three feature maps from the ultrasound original RF signal, and 1,050 radiomics features are extracted from the three feature maps; second, we extracted 37 spectral features from the normalized frequency spectrum after Fourier transform.

**Results:**

We use LASSO regression as the method for feature selection; moreover, we use support vector machine (SVM) for classification 10-fold cross-validation for examining the classification performance of the SVM. An AUC (area under the receiver operating characteristic curve) of 0.84 was obtained on 71 subjects.

**Conclusions:**

Our method is feasible to detect prostate cancer based on the ultrasound RF signal with superior classification performance.

## Introduction

Prostate cancer is one of the most common cancers among men worldwide, accounting for about 21% of all male cancers, and the fatality rate is about 8% (1), which ranks sixth among all male cancers. In 2019, there were an estimated 1.276 million new cases of prostate cancer worldwide and about 359,000 patients died of prostate cancer (2). For the diagnosis of prostate cancer, digital rectal examination (DRE) can only identify tumors in the posterior-surrounding area, so it cannot detect tumors that are located in the anterior periphery, central area, and transition area (3). Moreover, small tumors cannot be detected by palpation. Prostate-specific antigen (PSA) test based on blood tests is accompanied by a high risk of overdiagnosis and overtreatment (4). Magnetic resonance imaging (MRI) can provide functional tissue and anatomical information. T1-weighted imaging, T2-weighted imaging, diffusion-weighted imaging, and dynamic contrast-enhanced (DCE) multi-parameter MRI are commonly used in prostate diagnosis (5). However, studies have shown that MRI is also equally challenged in detecting prostate cancer, because generally MRI did little to predict the probability of either capsular invasion, seminal vesicle invasion, or lymph node metastasis (6).

Ultrasound RF signals contain richer tissue acoustic information than ultrasound images and have become a research area of interest in ultrasound tissue characterization in recent years. Khojaste and Imani (7) presented a feasibility study on differentiating between lower- and higher-grade prostate cancer using ultrasound RF time-series data. In the leave-one-out cross-validation strategy, an area under the receiver operating characteristic curve (AUC) of 0.78 has been achieved, and the overall sensitivity and specificity were 79% and 78%, respectively. Feleppa et al. (8) reported prostate tissue characterization methods that combine a set of features extracted from a spectrum analysis of RF signals with clinical data and used neural networks for classification and required results up to 80% in accuracy. Lin et al. (9) solved the problem of targeting and monitoring breast cancer with neural-network classification based on a spectrum analysis of ultrasound RF signals, and the AUC reached 0.804. However, in existing research, only some traditional texture features or some general spectral features were used (10, 11). Our method not only uses the spectral characteristics of the RF signal itself but also utilizes high-throughput spatial features from feature maps calculated from the RF signal; moreover, our method requires no additional equipment, and the selection of the region of interest is as simple as drawing a rectangular box within the lesion. Rectangular box size selection and placement tasks are much less subjective than manually drawing lesion outlines.

## Material and methods

### Data acquisition

Clinical data were collected from the Ultrasound Department, Peking University Third Hospital. The total 71 cases included 42 malignant cases and 29 benign cases.

The IC5-9D frequency conversion intracavitary probe of the LOGIQ E9 ultrasound diagnostic apparatus (GE Healthcare, USA) was used to perform transrectal prostate scanning and obtain the patients’ RF data. For patients who require prostate puncture, RF data were collected before puncturing the corresponding area of each tissue strip. The specific operation steps are depicted as follows: first confirming the puncture site, keeping the probe stable, turning on the “Collect RF Data” button which stays for 3 s, again clicking “Collect RF Data” to get the RF data of the area, puncturing the corresponding area, and then saving the regular gray-scale image. The puncture equipment uses BARD (Bard Peripheral Vascular, USA) disposable automatic puncture biopsy and an 18-G, 20-cm biopsy needle. The biopsy needle sampling slot is 2.2 cm long.

For healthy young patients, the RF data of four slices of the base, middle, apex, and mid-sagittal plane of the prostate were obtained. The final pathological results were not obtained for this part of the patients.

### Feature extraction

In total, we extracted three types of features: 1) time domain features, 2) frequency domain features, and 3) feature map. The first two types of features reflect the characteristics of time dimension and frequency dimension, respectively; because the RF signal is a time-series signal, the characteristics of time and frequency dimension are complementary. On the other hand, the RF signal also contains very rich features of ultrasonic attenuation, scattering, etc. Therefore, features only extracted from the time domain and frequency domain cannot guarantee the completeness. Therefore, we transformed the RF signal into the feature map, respectively, from the statistical distribution of signals, the energy attenuation, and non-linear signal (signal asymmetry) to extract the feature map. Combined with the high-throughput feature extraction method of radiomics, the features of these feature maps are extracted.

The general procedure can be roughly described as follows: acquire the ultrasound echo RF signal of the tissue, analyze and read the ultrasound RF file, extract a certain frame of ultrasound RF data, perform the Hilbert transformation on the extracted ultrasound RF data, display the ultrasound B-mode image, and select the region of interest (ROI) on the B-mode image. For the classification of cancerous and normal classes, we use some spectral features and a group of traditional texture features and extract them from the discrete Fourier transform (DFT) for the RF signals. For spectral features, spectral analysis is conducted using the Fourier transform of the Hamming-windowed 1-mm by 1-mm patch-wise RF sequences, where the Hamming window is used to suppress the high-frequency components at the start and end of the RF signal.

According to the center frequency of 4 MHz of the probe, a frequency range of 0–25 MHz is used, covering the vibration frequencies of 4, 8, 12, 16, 20, and 24 MHz. We divide the entire frequency band into seven sub-bands of [0, 1.5], [1.5, 6.5], [6.5, 10.5], [10.5, 14.5], [14.5, 18.5], [18.5, 22.5], and [22.5, 25] MHz, where the lowest and highest sub-bands have a frequency interval of 1.5 and 2.5 MHz, respectively, whereas the middle five sub-bands have a frequency interval of 4 MHz. The rationale of this division is as follows: first, the low-frequency components are more susceptible to white noise and thus are not considered. Second, the six vibration frequencies fall into the centers of the respective sub-bands instead of sub-band edges. Third, the magnitude of the frequency spectra is significantly lower at higher harmonics (16, 20, 24 MHz) compared with the lower harmonics (4, 8, 12 MHz).

From the estimated power spectrum, the following features (12) were computed:

Signal power—it gives the power of the signal in the frequency band


(1) $$\sum_{f=f_{u}}^{f=f_{l}}P\left(f\right)\Delta f$$

where *P*(*f*) is the estimated power spectral density (PSD) and *f_u_* and *f_l_* represent the upper and lower limits of the given frequency band, respectively.

Spectral centroid—it represents the weighted average frequency of the area under the PSD of the specified frequency band. Therefore, if there is a main peak, this feature can identify its location.


(2) $$\frac{\sum_{f=f_{u}}^{f=f_{l}}fP\left(f\right)\Delta f}{\sum_{f=f_{u}}^{f=f_{l}}P\left(f\right)\Delta f}$$

Spectral bandwidth—it represents the weighted average of the squared distances between the different frequency components and the spectral centroid (SC), where the weight is an estimate of the PSD at each frequency.


(3) $$\frac{\sum_{f=f_{u}}^{f=f_{l}}{\left(f-SC\right)}^{2}P\left(f\right)\Delta f}{\sum_{f=f_{u}}^{f=f_{l}}P\left(f\right)\Delta f}$$

Spectral flatness—also called tonality coefficient; it quantifies the pitch of the signal, not the noise. For a completely flat power spectrum, i.e., white noise, it evaluates to 1. Spectral flatness is calculated as the ratio of the geometric mean to the arithmetic mean of the PSD, as shown in the following equation:


(4) $$\frac{{\left(\prod_{f=f_{u}}^{f=f_{l}}P\left(f\right)\right)}^{\frac{1}{f_{u}-f_{l}}}}{\frac{1}{f_{u}-f_{l}}\sum_{f=f_{u}}^{f=f_{l}}P\left(f\right)\Delta f}$$

Crest factor—it has a similar definition to signal tonality. Functionally, it can be used to distinguish between wideband signals (with a smaller crest factor) and narrowband signals (with a larger crest factor).


(5) $$\frac{\text{max}\left(P\left(f\right)\right)}{\frac{1}{f_{u}-f_{l}}\sum_{f=f_{u}}^{f=f_{l}}P\left(f\right)\Delta f}$$

Each RF time series is a discrete signal of length N. After removing the mean and zero padding of the length of the time series, we use the fast Fourier transform (FFT) algorithm as implemented in MATLAB, to estimate the frequency spectrum of the RF time series and then to normalize the spectrum. We fit a regression line to values of the spectrum (versus normalized frequency). The intercept (S1) and the slope (S2) of this line are used as two more features (13). A total of 37 spectral features are extracted for each patient.

We extract high-throughput spatial features from feature maps calculated from the RF signal and use the following three feature maps: Nakagami distribution mean diagram (NDM), direct energy attenuation diagram (DEA), and RF signal skewness intensity diagram (RF-I).

The Nakagami statistical model, as a great model which simulates the shape of the probability density function of the backscattered echoes, is general enough to describe a wide range of the scattering conditions in medical ultrasound (14), including pre-Rayleigh, Rayleigh, and post-Rayleigh distributions. The pdf *f*(*r*) of the ultrasound backscattered envelope *R* under the Nakagami statistical model is given by (15):


(6) $$f\left(r\right)=\frac{2m^{m}r^{2m-1}}{\text{Γ}\left(m\right){\text{Ω}}^{m}}\exp\left(-\frac{m}{\text{Ω}}r^{2}\right)U\left(r\right)$$

where $m=\frac{{\left(E\left[r^{2}\right]\right)}^{2}}{Var\left[r^{2}\right]}$ , Ω= *E*[*r* ^2^] The Nakagami parameter *m* is a shape parameter that determines the statistical distribution of the ultrasound backscattered envelope (16), and m is obtained from


(7) $$m=\frac{{\left[E\left(R^{2}\right)\right]}^{2}}{E{\left[R^{2}-E\left(R^{2}\right)\right]}^{2}}$$

The Nakagami image is based on the Nakagami parameter map, which is constructed by using a local sliding window to process the raw envelope image. This involves first using a window within the envelope image to collect the local backscattered-signal envelopes for estimating the local Nakagami parameter (m), which is assigned as the new pixel located in the center of the window. This step is then repeated with the window moving throughout the entire envelope image in steps of one pixel, which yields the Nakagami image as the map of m values. The window size determines the resolution of the Nakagami image: using a smaller window will improve the resolution, but it will also yield fewer envelope data points, which can lead to unstable estimates of m (overestimation).

DEA means the scatterers absorb or scatter and attenuate, offset, or even exceed the increase in the intensity of the scatter signal caused by the increase in the concentration of the scatterers in the region of interest, resulting in the attenuation of the middle and deep scatter signals in the ultrasound image. The specific algorithm is as follows: set length segment = 64 (in pixels, the same below), seg interval = 16, sample rate = 3.2*10^7^ Hz, sound speed = 154,000 cm/s. For the direct energy coefficient (DEC) at a certain point on a certain beam of the RF input signal, first, this point is taken as the starting point and the length segment as the length of this RF signal for fast Fourier transform (FFT), and then the total energy E0 of this segment of the signal is calculated. Then, fast Fourier transform is performed on this segment of the RF signal starting from the point at a seg interval from this point, where the length segment is the length, and then the total energy E1 of this segment of the signal is also calculated. Then, the DEC at this point is defined as:


(8) $$DEC=\frac{10lg\left(\frac{E0}{E1}\right)}{\frac{S1*S2}{2S3}}$$

where S1 is the segment interval, S2 is the sound speed, and S3 is the sample rate.

After doing the same calculation program for all beams, the DEC of all points can be calculated to form a DEA feature map.

RF signal skewness intensity diagram (17): generally, RF signal intensity includes the average value, standard deviation, skewness, and kurtosis. Skewness is a measure of the direction and degree of skewness of statistical data distribution and a numerical feature of the degree of asymmetry of statistical data distribution. The specific calculation formula is shown below:


(9) $$Skew\left(X\right)=E\left[{\left(\frac{X-E\left(X\right)}{\sigma \left(X\right)}\right)}^{3}\right]=\frac{k_{3}}{{\left(\sigma \left(X\right)\right)}^{3}}=\frac{k_{3}}{k_{2}^{\frac{3}{2}}}$$

where *E*(*X*) is the mean, *σ*(*X*) is the standard deviation, E is the expectation operator, *k*_3_ is the third central moment, and *k_i_* is the *i*th cumulants.

For each feature map, 16 histogram features, 54 texture features, and 280 wavelet features of four different frequency sub-bands are extracted. The detailed introduction of radiomics features is defined in the appendix.

The specific description of the 16 histogram features and four different types of texture features are presented in **Table 1**. A total of 1,050 features are extracted for each patient from three ultrasound feature maps.

**Table 1:** All extracted features.

Feature type	Feature name	Feature amount
**Histogram**	1) Energy, 2) entropy, 3) kurtosis, 4) mean, 5) mean absolute different (MAD), 6) media, 7) range, 8) uniformity, 9) variance, 10) root mean square (RMS), 11) skewness, 12) deviation, 13) kurtosis, 14) mean, 15) variance, 16) skewness	16
**GLCM**	17) Energy, 18) entropy, 19) dissimilarity, 20) contrast, 21) inversed difference, 22) correlation, 23) homogeneity, 24) autocorrelation, 25) cluster shade, 26) cluster prominence, 27) maximum probability, 28) sum of squares, 29) sum average, 30) sum variance, 31) sum entropy, 32) difference variance, 33) difference entropy, 34) information measures of correlation1, 35) information measures of correlation2, 36) maximal correlation coefficient, 37) maximal correlation coefficient, 38) inverse difference normalized, 39) inverse difference moment normalized	23
**GLRLM**	40) Short-run emphasis, 41) long-run emphasis, 42) gray-scale non-uniformity, 43) run-length non-uniformity, 44) run percentage, 45) low gray-level run emphasis, 46) high gray-level run emphasis, 47) short-run low gray-scale emphasis, 48) short-run high gray-scale emphasis, 49) long-run low gray-level emphasis, 50) long-run high gray-level emphasis, 51) gray-level variance, 52) run-length variance	13
**GLSZM**	53) Small zone emphasis, 54) large zone emphasis, 55) gray-scale non-uniformity, 56) zone-size non-uniformity, 57) zone percentage, 58) low gray-level zone emphasis, 59) high gray-level zone emphasis, 60) small zone low gray-level emphasis, 61) small zone high gray-level emphasis, 62) large zone low gray-level emphasis, 63) large zone high gray-level emphasis, 64) gray-level variance, 65) zone-size variance	13
**NGTDM**	66) Coarseness, 67) contrast, 68) busyness, 69) complexity, 70) strength	5
**wavelet features**	The 70 features after wavelet transform are completely consistent with those in the table above, except that they correspond to four sub-bands with different frequencies.	280
Total number of all features: 350		

In the “Results” section, we extract the features proposed in two previous papers and compare the results with our features. In Khojaste and Imani’s paper (7), it proposes 60 spectral features of RF time series based on SVM with a radial basis kernel function for the detection of prostate cancer. In Zheng and Lin’s study (18), they extracted three different dimensions of parameters of the ROI, namely, time domain, frequency domain, and fractal dimension (FD), for cervical cancer diagnosis. Fourteen spectrum characteristic parameters were extracted, of which spectral fractal dimension (SFD) and Higuchi FD belong to FD parameters. Slope, intercept, mid-band fit, and four features which are the average value of the normalized spectrum in four quarters of the frequency range are the frequency domain parameters. Fuzzy entropy, kurtosis, peak, cross zero count, and cross zero standard deviation (Std) are the time-domain parameters.

### Feature selection

In order to enhance the generalization ability of the model, reduce overfitting, and enhance the understanding of features and eigenvalues, we need to choose a feature selection method to reduce the number of features to prevent dimensional disasters. Since the total number of features extracted is large (>1,000) and our purpose is to select a specific feature subset for subsequent research, a large number of features either have multicollinearity or have little effect on classification, which need to be eliminated in our subsequent research; that is, sparse models need to be generated. For this reason, we choose the Least Absolute Shrinkage and Selection Operator (LASSO) logistic regression algorithm as our feature selection method (19). In our paper, LASSO is used for initial feature selection. Since three types of features are extracted from the RF signal, the feature dimension of each case is high. For classification, some features are redundant and not conducive to the final classification decision. Therefore, the classical LASSO regression is used for initial feature selection, and the features with high redundancy and little contribution to classification have been removed. LASSO is a contraction estimation method based on the idea of feature dimension reduction, and it obtains a relatively refined model by constructing a penalty function. Using it to compress some coefficients, while setting some coefficients to zero, retains the advantage of subset shrinkage and is a biased estimate for dealing with complex collinear data.

The statistical correlation between some of our proposed features is fairly high. For example, the correlation coefficient of S1 and S5 is 0.91 in our dataset, and this is due to the fact that both of these values are highly affected by the pattern of the low-frequency band of the spectrum. That is why we choose LASSO regression as a feature selection method to avoid the curse of dimensionality.

### Classification and validation

We use the support vector machine (SVM) for classification based on the proposed features and use 10-fold cross-validation for examining the classification performance of the SVM. In the 10-fold cross-validation strategy, we repeat the 10-fold cross-validation process 50 times, each time with a different partitioning of the data in 10 parts. We report the mean and standard deviation of the outcomes of these 50 trials. After obtaining the probability that the input data belong to a certain class, these predicted probabilities are arranged from small to large, and then the classification threshold is set to different probability values in the [0, 1] interval and the sensitivity and specificity calculated at this time. Values of sensitivity plotted versus specificity create the ROC curve.

## Results

### Patient baseline information

We collected the clinical data from the Ultrasound Department, Peking University Third Hospital. The total 71 cases included 42 malignant cases and 29 benign cases. We counted the baseline information of all cases, which includes age, PSA information, and TRUS information. The statistical results are presented in **Table 2**.

**Table 2:** Baseline information of all 71 cases.

Demographic or clinical characteristic	Average value (std)		P-value
	Malignant	Benign	
**Age (year)**	70.29±8.16	67.43±8.89	0.196
**tPSA**	24.32±26.44	14.26±11.93	0.063
**fPSA**	3.43±5.22	2.66±4.03	0.528
**TRUS transverse diameter (cm)**	4.87±0.98	5.41±0.82	0.024
**TRUS vertical diameter (cm)**	4.27±0.96	5.01±1.31	0.015
**TRUS anteroposterior diameter (cm)**	3.59±0.86	4.29±1.19	0.011
**Prostate volume (cm^3^)**	44.20±44.65	68.22±49.36	0.051

### Study results

**Figure 1:**
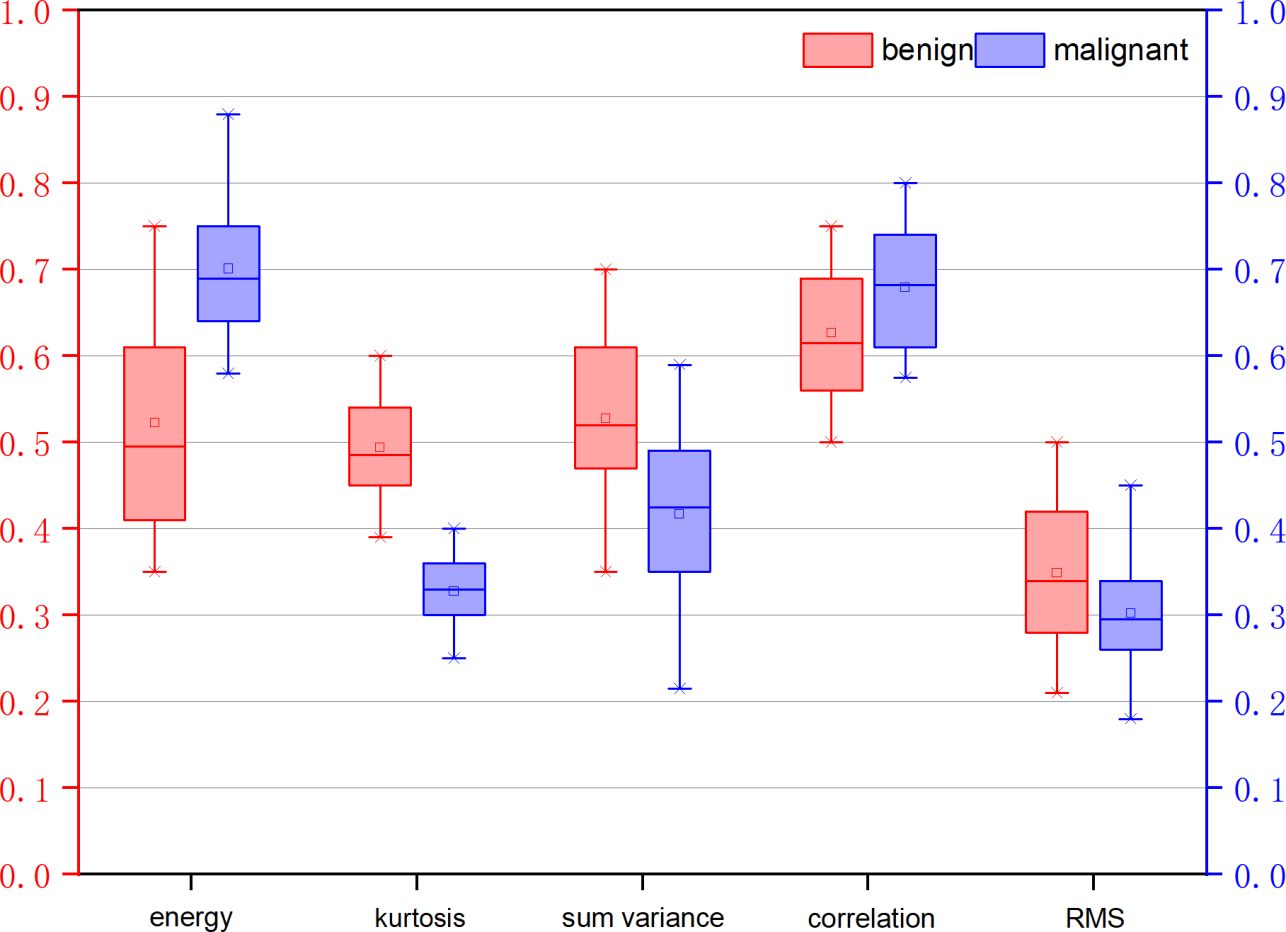
Normalized feature value of features selected from the feature map direct energy attenuation diagram (DEA).

**Figure 2:**
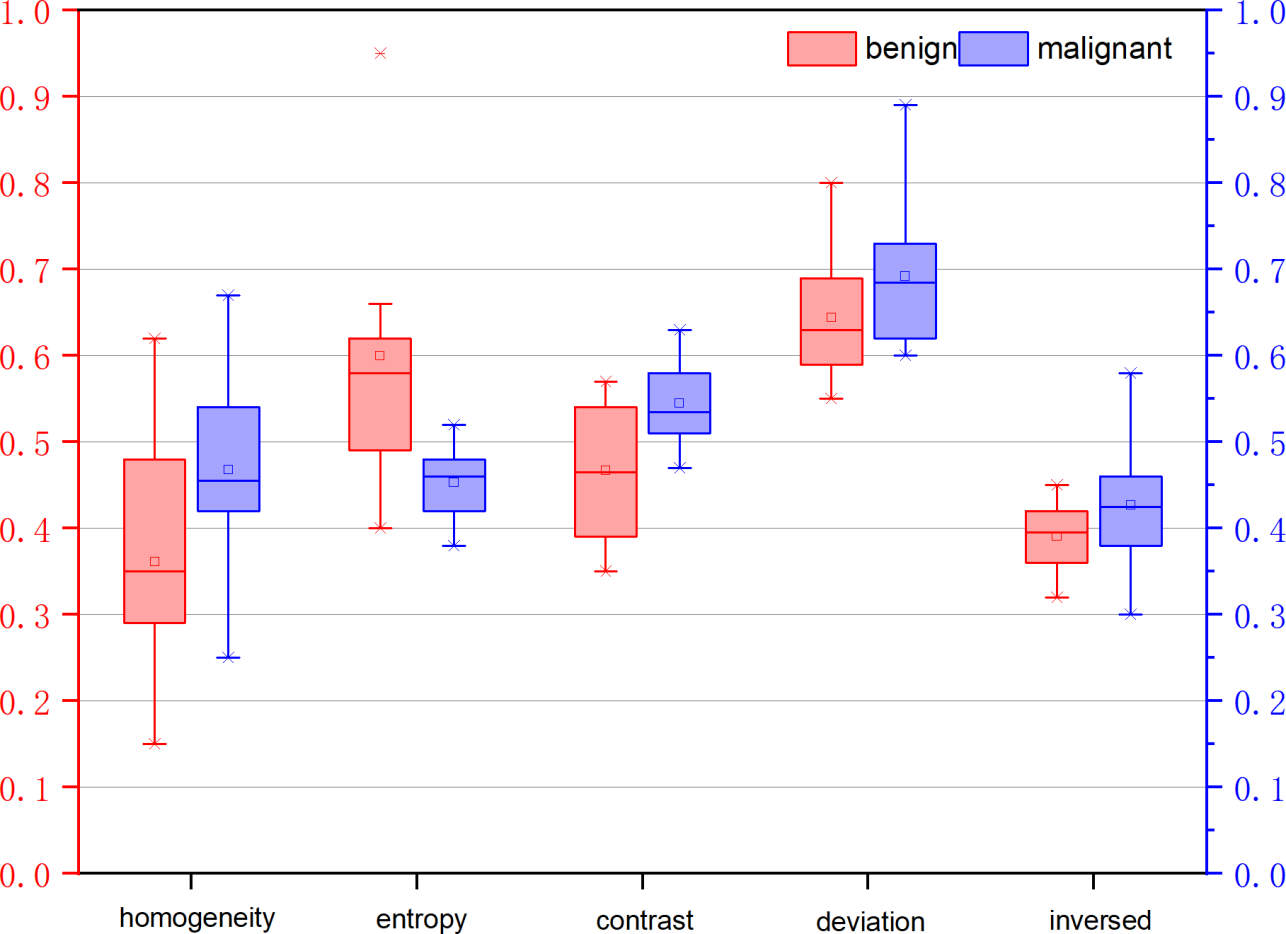
Normalized feature value of features selected from the feature map Nakagami distribution mean diagram (NDM).

**Figure 3:**
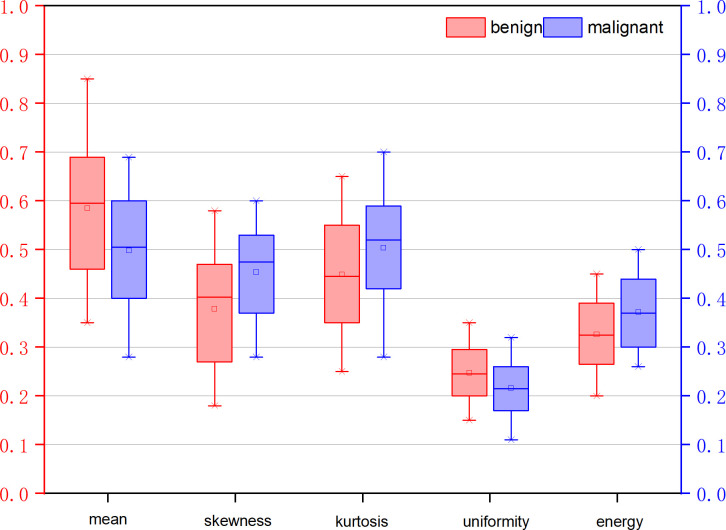
Normalized feature value of features selected from the feature map RF signal skewness intensity diagram (RF-I).

**Table 3** illustrates the statistical differences of the intercept, slope, and average value of five-spectral-feature band in ROIs belonging to benign and malignant classes. Except for the slope and intercept, each sub-band takes five identical features. It is inconvenient to list all 37 features here, so the average value of all sub-bands is taken for these five features. Because the sample size of the data is small (<100), here we choose the T-test to prove the statistical significance. The smaller the P-value, the higher the possibility of a difference between benign and malignant classes. When the P-value is less than 0.05, there is a significant statistical difference between them. According to the table, the overall best feature appears to be the three-dimensional feature vector of signal power, spectral flatness, and crest factor. The P-value of the intercept is extremely close to 0.01, whereas the P-value of the spectral bandwidth is significantly higher than 0.01. For texture features, as the number of extracted features reaches as many as several hundred, only the final filtered features can be statistically analyzed. The selected texture features are as follows: DEA—histogram energy, histogram kurtosis, gray-level concurrence matrix (GLCM) sum variance; NDM—histogram entropy, GLCM homogeneity, neighborhood gray-tone difference matrix (NGTDM) contrast; RF-I—histogram mean, histogram skewness, histogram kurtosis. The normalized feature value of these features is presented in **Figures 1**–**3**.

**Table 3:** T-test results to prove the statistical significance between malignant and benign classes.

Parameter	Average value (std)		P-value	T value
	Malignant	Benign		
**Intercept**	0.21±0.048	0.19±0.03	0.010	-3.390
**Slope**	-0.26±0.06	-0.24±0.04	0.049	1.987
**Signal power**	258.4±10.06	223.6 ± 9.85	<0.010	-5.268
**Spectral centroid (MHz)**	4.24±0.57	4.35 ± 0.74	0.034	1.259
**Spectral bandwidth (MHz)**	2.61 ± 0.24	2.53 ± 0.31	0.065	2.027
**Spectral flatness**	0.87 ± 0.09	0.68 ± 0.07	<0.010	-4.812
**Crest factor**	1.96 ± 0.34	1.72 ± 0.25	<0.010	-3.681

**Figure 4:**
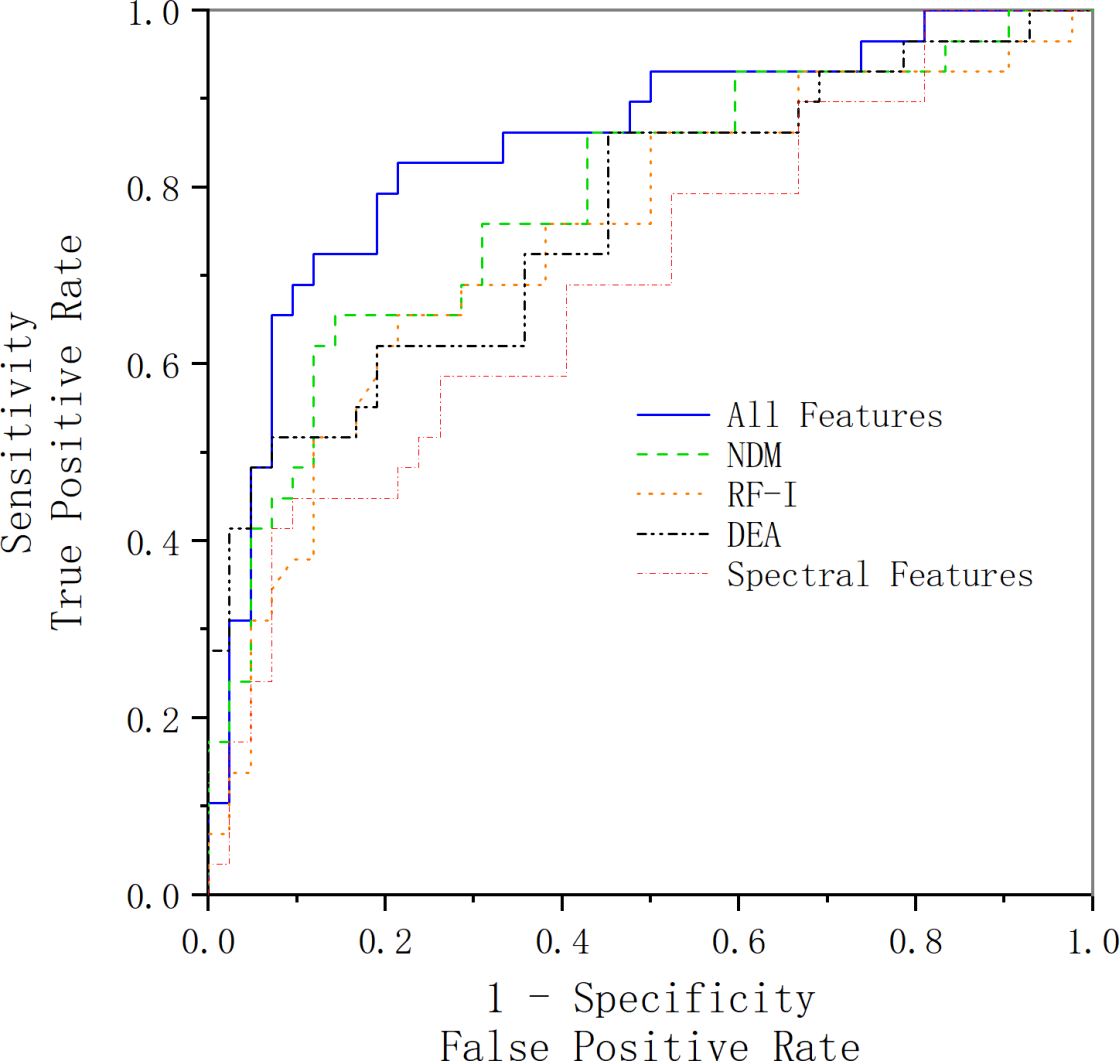
ROC curve acquired using the selected feature vector along with an SVM classifier. The blue line represents the ROC curve acquired using all features we have extracted and selected, the green line represents the ROC curve acquired using the features extracted from the NDM feature map, the black line represents the ROC curve acquired using the features extracted from the DEA feature map, the orange line represents the ROC curve acquired using the features extracted from the RF-I feature map, and the red line represents the ROC curve acquired using the spectral features.

**Table 4** shows the classification results. The ROC curve for a set of features is illustrated in **Figure 4**. However, it should be noted that although we successfully detected the examined cancerous ROIs in all cases, the false-positive rate is relatively low (specificity of 79.7%). The relatively low specificity of the results might be an indication of insufficient training of the classifier on the benign data that can potentially be addressed by increasing the number of benign ROIs. Moreover, it should be noted that an alternative theory explains that the low specificity is the presence of “different types of benign tissue” in the prostate. It is a well-known fact that prostate tissue can exhibit benign histological variations (including but not limited to benign prostatic hyperplasia). These conditions are specifically common in patients with prostate cancer and are characterized by cellular microstructures that do not match either normal or cancerous classes.

**Table 4:** The classification results of different feature sets.

	AUC (std)	ACC (std)	SENS (std)	SPEC (std)
**All features**	0.842 (0.12)	0.821 (0.08)	0.808 (0.06)	0.797 (0.04)
**DEA**	0.720 (0.09)	0.691 (0.06)	0.662 (0.08)	0.696 (0.07)
**NDM**	0.748 (0.07)	0.702 (0.06)	0.684 (0.09)	0.725 (0.06)
**RF-I**	0.703 (0.12)	0.659 (0.04)	0.621 (0.06)	0.608 (0.03)
**Intercept**	0.639 (0.08)	0.605 (0.03)	0.616 (0.03)	0.598 (0.04)
**Slope**	0.608 (0.09)	0.583 (0.08)	0.596 (0.04)	0.562 (0.06)
**Signal power**	0.757 (0.10)	0.714 (0.05)	0.725 (0.06)	0.701 (0.06)
**Spectral centroid**	0.708 (0.09)	0.682 (0.06)	0.679 (0.05)	0.654 (0.05)
**Spectral bandwidth**	0.712 (0.08)	0.695 (0.09)	0.662 (0.05)	0.687 (0.07)
**Spectral flatness**	0.749 (0.11)	0.726 (0.06)	0.704 (0.04)	0.716 (0.06)
**Crest factor**	0.732 (0.13)	0.708 (0.05)	0.717 (0.05)	0.689 (0.07)

**Figure 5:**
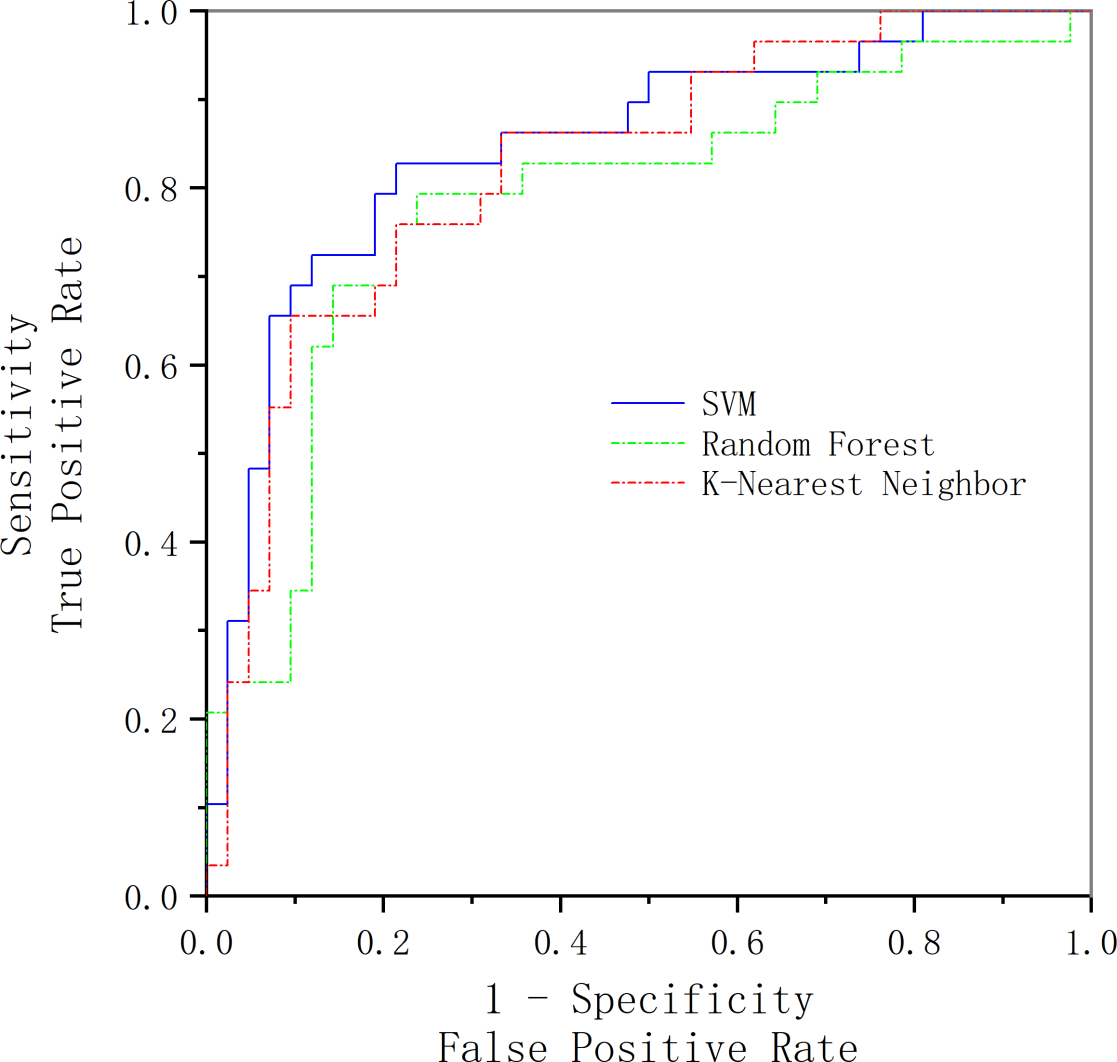
Comparison of classification results of three classification methods.

In order to verify the accuracy of our selection of SVM as the classification method, we select SVM, random forest, and K-nearest neighbor as the classification methods and compared the final classification results. The comparison results are shown in **Figure 5**. The results show that the AUC of SVM, random forest, and K-nearest neighbor classification results are 0.842, 0.825 and 0.785, respectively. The comparison results show that SVM is still the best classification method for small samples, whereas random forest and K-nearest neighbors, which are better at dealing with high-dimensional data and multi-classification problems, perform slightly worse.

**Figure 6:**
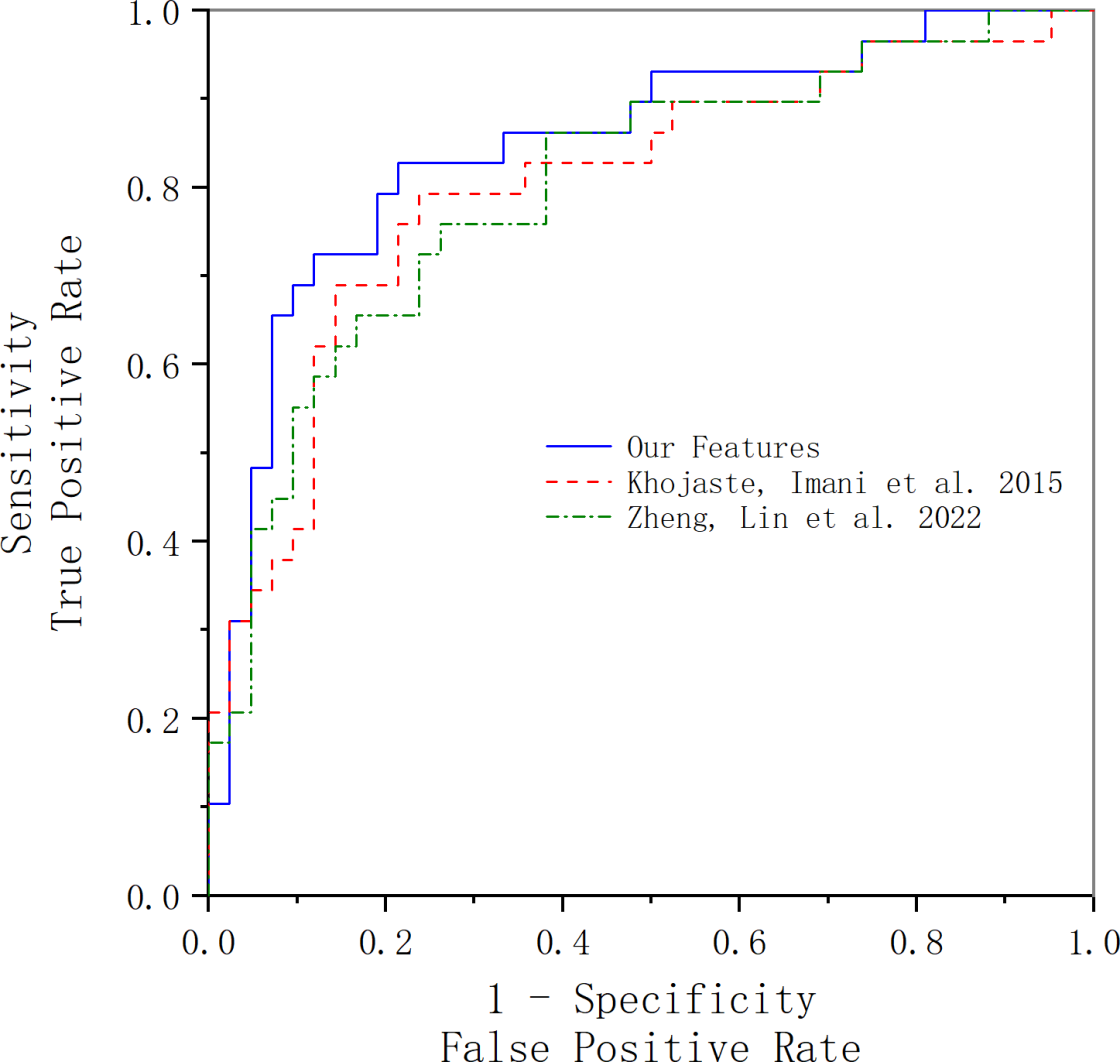
ROC curve acquired using our features and Khojaste and Imani’s features. The blue line represents the ROC curve acquired using our features; the red line represents the ROC curve acquired using Khojaste and Imani’s features; the green line represents the ROC curve acquired using Zheng and Lin’s features.

In this part, we compare our method with others. In Khojaste and Imani’s paper (7), we use their features presented in their paper for our data; the comparison of the results is presented in **Figure 6** and **Table 5**. Compared with their frequency domain features, wavelet transform features and traditional texture features comprehensively reveal more tumor tissue details. Therefore, our feature amount is evidently more than theirs. Furthermore, our classification results are slightly better than theirs. Because of the algorithm’s complexity and time consumption, our method could still be used to accurately characterize prostate cancer and distinguish benign and malignant tumors.

**Table 5:** The classification results of our features and Zheng and Imani’s features.

	AUC (std)	ACC (std)	SENS (std)	SPEC (std)
**Our features**	0.842 (0.12)	0.821 (0.08)	0.808 (0.06)	0.797 (0.04)
**Khojaste, Imani et al.** (7)	0.809 (0.07)	0.785 (0.07)	0.721 (0.09)	0.796 (0.06)
**Zheng, Lin et al.** (18)	0.796 (0.09)	0.784 (0.10)	0.759 (0.05)	0.742 (0.08)

In Zheng and Lin’s study (18), we used all their features with our data for comparison of results, and the comparison of the results is presented in **Figure 6** and **Table 5**. The frequency domain and FD of ultrasound RF time-series signals are parameters that reflect the complexity, roughness, and irregularity of the tissue surface, which may overlap with traditional gray-scale ultrasound imaging in identifying tissues. However, compared with our texture and wavelet features, the time-domain features’ statistic difference between benign and malignant tissues is not significant, which may be the main reason for lower results compared with ours.

## Discussion

This study aims to detect prostate cancer based on the ultrasound RF signal, utilizing low-dimensional features in the frequency domain and high-throughput features in the spatial domain. For each patient, we extracted 37 spectral features in the frequency domain and extracted a total of 1,050 features (included histogram features, texture features, and wavelet features) from three ultrasound feature maps (DEA, NDM, RF-I) in the spatial domain. After using LASSO as the feature selection method, we used SVM for classification based on the proposed features and use 10-fold cross-validation for examining the classification performance of the SVM. In the results section, a T-test for spectral features showed that there is a significant statistical difference between these spectral features. Signal power, spectral flatness, and the crest factor are the overall best feature vector. With the use of SVM and 10-fold strategy classification, the selected features provide a diagnostic method with the area under the ROC curve of 0.84 and accuracy of 0.82.

Compared with Khojaste and Imani’s paper (7), both papers have extracted spectral features, but the feature amount of theirs (60 spectral features) is much less than ours. However, with the utilization of wavelet transform features and traditional texture features, our features could comprehensively reveal more tumor tissue details. Thus, our classification performance is better in comparison with theirs (AUC: 0.8, ACC: 0.78). Compared with Zheng and Lin’s study (18), they extracted FD features in addition to time domain features and frequency domain features, having good sensitivity and specificity for identifying cervical cancer. However, as to our data, no statistically significant differences about the FD features were found among benign and malignant cases with different degrees of differentiation. This may indicate that the FD features are not effective enough for identifying prostate cancer; also, the FD feature is also a kind of texture feature, which may overlap with our extracted texture features.

Currently, conventional B-mode ultrasound is the standard means of imaging the prostate for guiding prostate biopsies and planning radiotherapy. Yet, B-mode images essentially do not allow visualization of cancerous lesions of the prostate. Our paper provides a non-invasive method to detect prostate cancer and makes full use of the original RF signal information missing from the B-mode ultrasound, providing a reliable reference for clinicians to identify prostate cancer.

However, since there is no gold standard for pathological areas in the ultrasound gray-scale images of the original data provided by the hospital, and the position of the probe in some gray-scale images is extremely inconspicuous, it is difficult to obtain accurate ROI, so this study sets a square in the parenchymal region of the prostate near the needle as ROI without a precise ROI outline, which may lead to poor results. Therefore, we plan to discover accurate ROI outline methods in subsequent research to further improve the results. Furthermore, the amount of case data in this study is not enough, and follow-up work will further collect data to enhance the stability of the results.

## Data availability statement

The original contributions presented in the study are included in the article/supplementary material. Further inquiries can be directed to the corresponding authors.

## Author contributions

TX, QW and JY contributed to conception and design of the study. WS and LC organized the database. TX wrote the first draft of the manuscript. TX, JY, and GW contributed to manuscript revision, read, and approved the submitted version.

## Appendix

**The detailed introduction of radiomics features**

The histogram features (also called first-order histogram features) describe features associated with the distribution of pixel intensities within the ROI, excluding their spatial interaction, and can be calculated by histogram analysis. These features can reflect the symmetry, uniformity, and local intensity distribution of the measured pixels. It usually includes some basic statistical features, including mean, median, minimum, maximum, standard deviation, skewness, and kurtosis. However, depending on the actual task, further complex statistical features can be designed.

The texture features (also called second-order histogram features) describe the intensity level of pixel spatial distribution. Image texture refers to the spatial variation that can be perceived or measured at the intensity level. It is regarded as a gray level, which is a visual perception of the synthesis of image local features. It includes gray-level co-occurrence matrix (GLCM), gray-level run-length matrix, GLRLM), gray-level size zone matrix (GLSZM), and neighborhood gray-tone difference matrix (NGTDM). GLCM is a matrix whose row number represents the gray value and cells contain the number of times the gray value is in a certain relationship (angle, distance), also known as the second-order histogram. The features computed on GLCM include entropy (second-order entropy, related to heterogeneity), energy (also defined as angular second moments, again describing image uniformity), contrast (its measure of local variation), homogeneity (a measure of image local gray-level equilibrium), dissimilarity, and correlation. The GLRLM is used to describe the number of consecutive neighbors *j* of a pixel value *i* of an image along a certain direction *θ*. A value in GLRLM is denoted as *D*(*i,j,θ*), where *i* is the pixel value or gray value, *j* is the continuous adjacent number, *θ* is the angle or direction, and generally the values of *θ* are 0°, 45°, 90°, and 135°. GLSZM is a matrix where the elements at the rows and columns store the number of regions with grayscale levels and sizes (connected pixels with the same grayscale level). GLSZM includes features that describe the distribution of small/large areas and low/high gray areas. NGTDM, whose *i*th term is the sum of the differences between the average values of all pixels with gray tone *i* and their surrounding pixels. The radiomics features of NGTDM include roughness, contrast, redundancy, complexity, and texture intensity.
